# The Co-Expression Pattern of p63 and HDAC1: A Potential Way to Disclose Stem Cells in Interfollicular Epidermis

**DOI:** 10.3390/ijms18071360

**Published:** 2017-06-26

**Authors:** Jung-Won Shin, Hye-Ryung Choi, Kyung-Mi Nam, Hyun-Sun Lee, Sung-Ae Kim, Hyun-Jae Joe, Toyama Kazumi, Kyoung-Chan Park

**Affiliations:** 1 Department of Dermatology, Seoul National University Bundang Hospital, 166 Gumi-ro, Bundang-gu, Seongnam-si, Gyeonggi-do 463-707, Korea; spellbound00@hanmail.net (J.-W.S.); 97874@snubh.org (H.-R.C.); mbn01@naver.com (K.-M.N.); hyhssky@gmail.com (H.-S.L.); 2 Department of Dermatology, Keimyung University School of Medicine, 56 Dalseong-Ro, Jung-Gu, Daegu 41931, Korea; skksasf@hanmail.net (S.-A.K.); joehyunjae@hanmail.net (H.-J.J.); 3 P&G Group, Kobe 658-0032, Japan; Toyama@png.com

**Keywords:** epidermal stem cell, HDAC1, interfollicular epidermis, stem cell niche, p63, suberoylanilohydroxamic acid (SAHA)

## Abstract

Stem cell markers of interfollicular epidermis (IEF) have not been established thus far. The aim of this study is to suggest a new way to disclose IFE-stem cells by combining the expression of histone deacetylases (HDAC) 1 and p63. Immunohistochemical staining of HDAC1 and p63 was performed in six normal human samples. Moreover, a skin equivalent (SE) model was treated with suberoylanilohydroxamic acid (SAHA, an HDAC inhibitor) to elucidate the role of HDAC1. Finally, rapidly adhering (RA) keratinocytes to a type IV collagen, which have been identified to represent epidermal stem cells, were subjected to Western blot analysis with antibodies against HDAC1. In normal samples, there was a minor subpopulation comprised of p63-positive and HDAC1-negative cells in the basal layers. The proportion of this subpopulation was decreased with age. In the SE model, SAHA treatment increased the epidermal thickness and number of p63-positive cells in a dose dependent manner. After SAHA treatment, the expression of differentiation markers was decreased, while that of basement membrane markers was increased. In a Western blot analysis, HDAC1 was not expressed in RA cells. In conclusion, the combination of p63-positive and HDAC1-negative expressions can be a potential new way for distinguishing epidermal stem cells.

## 1. Introduction

The epidermal stem cells (ESC) play a critical role in the maintenance and regeneration of cutaneous epithelial tissues. A stem cell niche is a specific microenvironment that interacts with stem cells and regulates the activities and fate of the residing stem cells [1,2]. In the epidermis, there are three distinct ESC niches: Bulge of the hair follicle (HF), base of the sebaceous gland, and basal layer of the interfollicular epidermis (IFE) [3,4,5]. In normal conditions, each discrete compartment behaves unipotently, replenishing its own respective tissue compartments. To date, epidermal stem cell researchers have mostly been focused on HF-stem cells, and as a result, little is known about IFE-stem cells. However, from scarcely available sources, we know that tissue homeostasis in mouse IFE is maintained by an independent stem cell population [6,7]. In the human skin, stem cells in the IFE are expected to play a more important role in skin tissue homeostasis. Moreover, these cells are thought to be dispersed along the basement membrane, which is a complex network of extracellular matrix (ECM) molecules interacting through integrins. This interaction between integrins and ECM may be important in shaping the epidermal stem cell niche [8].

In IFE, there exists a hierarchy consisting of slow-cycling stem cells and rapidly cycling transit amplifying (TA) cells. After a few rounds of division, TA cells differentiate to post-mitotic cells that are no longer able to proliferate and undergo a process of terminal differentiation [9,10,11,12]. Recently, there have been findings supporting that slow-cycling cells, not TA cells in IFE contribute to long-term tissue repair [3,13]. While markers for HF-stem cells are well established, i.e., CD 34 [14] in mice and CD200 [15] in humans, markers for stem cells in IFE have not yet fully been elucidated. The p63 protein is an essential regulator for epidermal development and maintenance [16,17,18,19,20] and has been used as an epidermal stem cell marker; regardless, it has also been expressed in the upper layers of the epidermis, suggesting that it may not be a specific stem cell marker. Moreover, despite having highly proliferative cells in the basal layer expressing high levels of α6 integrins, K5, and K14 [20], it is unclear which subsets in the population express these markers [21,22].

Histone deacetylases (HDACs) are a class of enzymes with an important role in epigenetic modification. They remove the histone acetyl groups from histones, resulting in the compaction of chromatin structure and transcriptional repression [23]. Previously, it has been shown that HDAC1 is associated with the strongest expression in the outer differentiating epidermal cell layer during the late developmental stages [16]. Moreover, a nuclear complex, including E2F-5 and HDAC1, has also been revealed to be involved in the permanent withdrawal from the cell cycle responding to differentiation stimuli [24].

In this study, we attempted to identify the IFE-stem cells based on the presence or absence of two markers, p63 and HDAC1.

## 2. Results

### 2.1. HDAC1 and p63 Expressions According in Normal Human Skins in Different Age Groups

**Figure 1:**
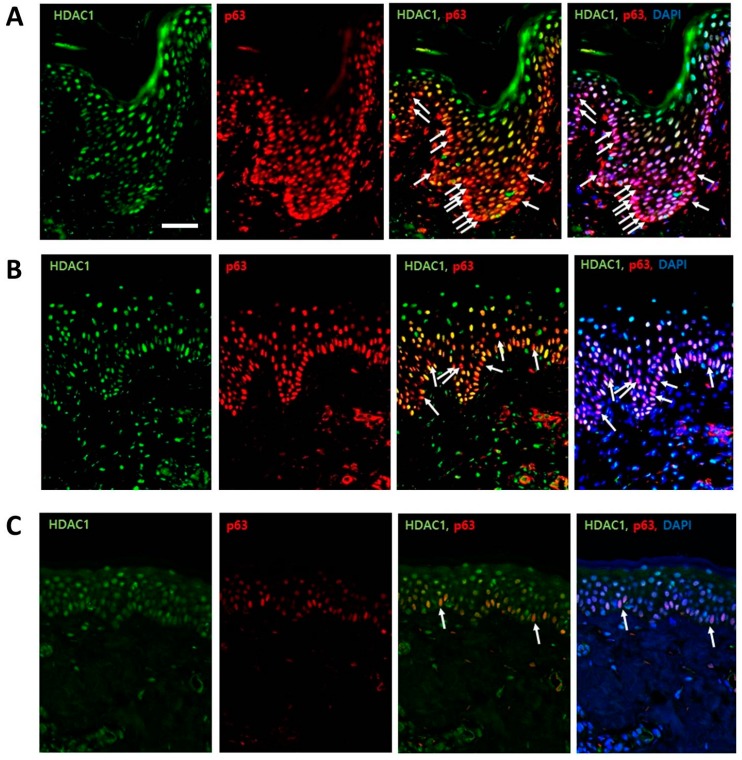
HDAC1 and p63 expressions in normal human skins. (**A**) HDAC1 and p63 expressions in the young-age group; (**B**) HDAC1 and p63 expressions in the middle-age group; (**C**) HDAC1 and p63 expressions in the old-age group. In a merged image, there was a minor subpopulation of cells with p63-positive/HDAC1-negative staining pattern in the basal layer (white arrow). The number of p63-positive and HDAC1-negative cells was decreased with age. (Green: HDAC1 staining, red: p63 staining, ×200, scale bar is 50 μm).

In all six human samples, HDAC1 and p63 showed a consistent pattern. HDAC1 was expressed throughout the epidermis, with a strong localization near the nuclei of the outer, differentiating cell layers. Some cells in the basal layer were completely negative for HDAC1 (Figure 1). In contrast, p63 was most strongly expressed in the basal layer, with moderate expression in the upper layer; the staining intensity decreased as the layer increased, and the cells in the granular layer were rarely stained. In a merged image, there was a subpopulation showing a p63-positive/HDAC1-negative staining pattern in the basal layer, and the number of these cells decreased gradually with age (white arrows, Figure 1).

**Figure 2:**
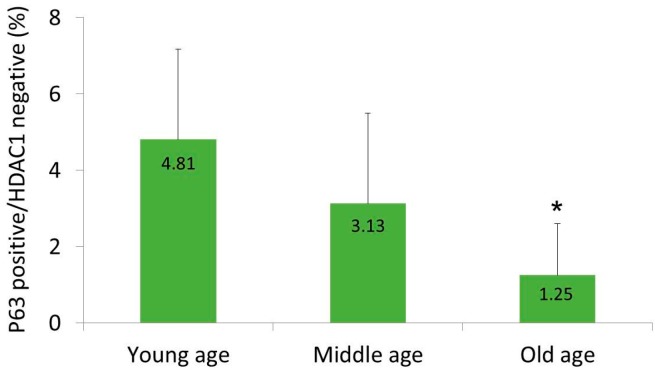
The proportion of p63-positive/HDAC1-negative cells in epidermis. Six areas were randomly selected in each sample and the average percentage was calculated. Statistical significance was calculated with the Mann-Whitney-*U* test, * *p* < 0.01 compared with the young-age and *p* < 0.05 compared with the middle-age group.

At 200× magnification, six fields with the same area were randomly chosen in each sample. Then, the percentages of epidermal cells with a p63-positive/HDAC1-negative staining pattern were calculated. In the young-age group, this subpopulation was occupying 4.81 ± 2.36% of the total epidermal cells (Figure 2); in the middle-age group, the portion of this subpopulation was lower, at 3.13 ± 2.36% (Figure 2); and in the old-age group, only 1.25 ± 1.35% of the epidermal cells showed a p63-positive/HDAC1-negative staining pattern (Figure 2). The average percentages of epidermal cells with p63-positive/HDAC1-negative staining pattern significantly decreased with age. In the old-age group, the percentage was significantly lower than in the young-age group (*p* < 0.01) and in the middle-age group (*p* = 0.028).

### 2.2. Reconstruction of the Skin Equivalent and the Effect of Suberoylanilohydroxamic Acid

**Figure 3:**
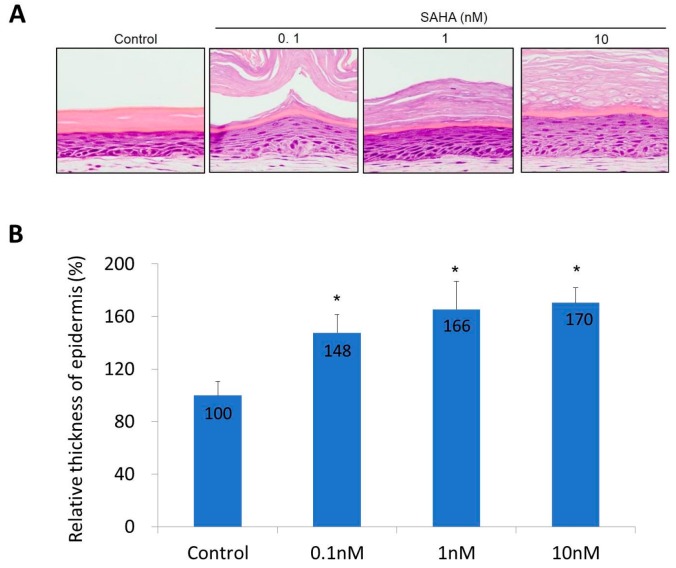
The change of epidermis after SAHA treatment in skin equivalent (SE). (**A**) H&E staining of SE; (**B**) The relative thickness of epidermis. Eight areas were randomly selected in each dose and the average relative thickness was calculated. Statistical significance was calculated with the Mann-Whitney-*U* test, * *p* < 0.01 compared with the control. This experiment was repeated twice and representative areas are shown.

The toxicity of SAHA was tested using a monolayer culture from normal human fibroblasts and keratinocytes. Cultured fibroblasts and keratinocytes were treated with increasing concentrations of SAHA, ranging from 0.1 to 10 nM for 24 h. The cell viability experiment showed that suberoylanilohydroxamic acid (SAHA) was not cytotoxic to both cells at concentrations of up to 10 nM. After this, the skin equivalent (SE) model was constructed and histological features were observed. Characteristic multi-layering and stratification of the epidermis was found. When SAHA was added, the epidermis of SE became thicker compared with the control SE. The basal layer cells became more cuboidal and the number of keratinocyte layers increased in a dose-dependent manner (Figure 3A). Eight areas were randomly selected in each dose and the relative thickness was calculated (Figure 3B). The epidermal thickness increased about 1.5 times in the 0.1 nM SAHA treated sample, and about 1.7 times in the 10 nM SAHA treated sample, compared with the control (*p* < 0.01). Since SAHA did not affect the proliferation of fibroblasts and keratinocytes, at concentrations of up to 10 nM, these results may come from an indirect effect of SAHA. The control and SAHA (0.1, 1, 10 nM) treated models were selected for further immunohistochemical analysis.

**Figure 4:**
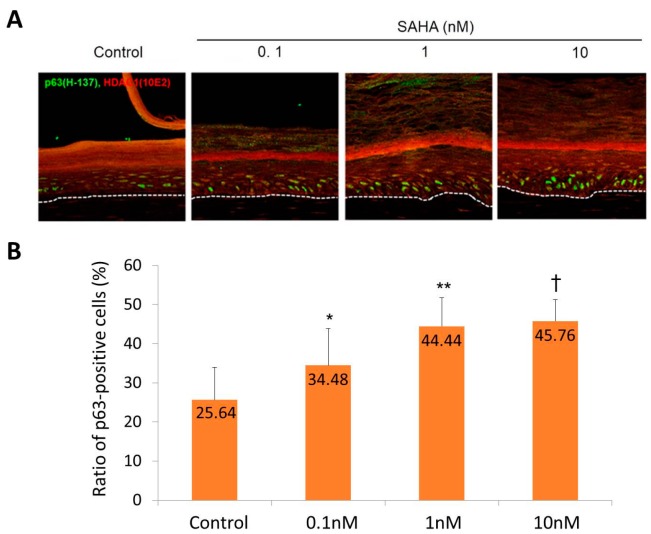
The p63 and HDAC1 staining in skin equivalents, (**A**) Confocal microscopic examination in SAHA treated samples (green; p63 staining, red; HDAC1 staining, ×200); (**B**) The ratio of epidermal cells with p63-positive staining in SAHA treated samples. Six circular areas were randomly selected and the percentage of p63-positive cells to total epidermal cells were calculated. Statistical significance was calculated with the Mann-Whitney-*U* test, * *p* < 0.05 compared with the control, ** *p* < 0.01 compared with the control, ^†^ *p* < 0.01 compared with the 0.1 nM. This experiment was repeated twice and representative areas are shown.

Confocal microscopic examination showed an increased number of p63-positive cells in SAHA treated samples (Figure 4A). The percentages of epidermal cells with p63-positive staining were 25.64 ± 7.43% in the control sample, 34.48 ± 8.37% in the 0.1 nM SAHA treated sample, 44.44 ± 6.55% in the 1 nM SAHA treated sample, and 45.76 ± 4.95% in the 10 nM SAHA treated sample (Figure 4B).

**Figure 5:**
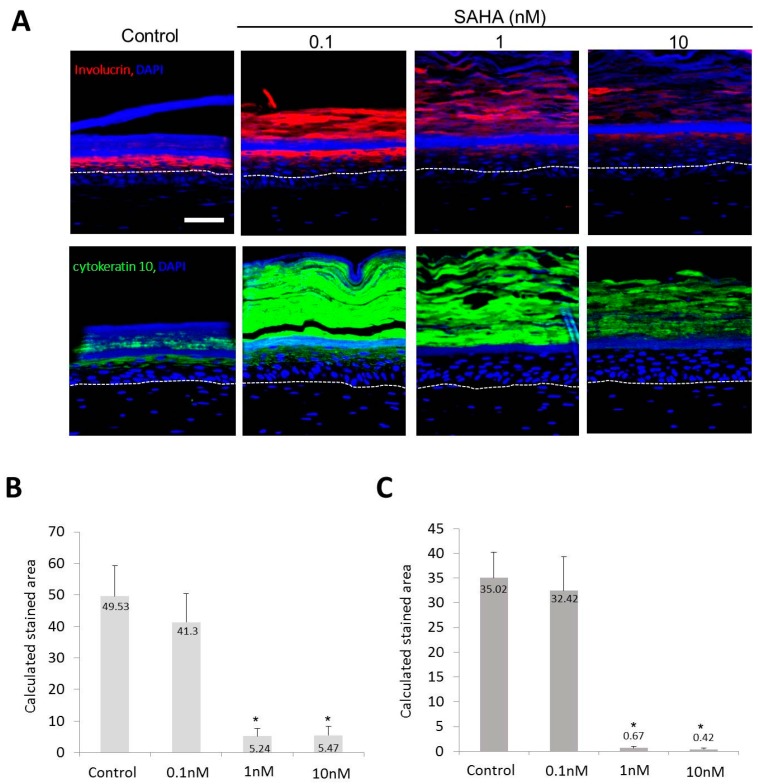
Expression of epidermal differentiation markers in SE. (**A**) Confocal microscopic staining (red; involucrin staining, green; cytokeratin 10 staining, ×200, scale bar is 50 μm); (**B**) Calculated stained area for involucrin after SAHA treatment; (**C**) Calculated stained area for cytokeratin 10 after SAHA treatment. Sis circular areas were selected and the average area stained above a certain intensity was determined. Statistical significance was calculated with the Mann-Whitney-*U* test, * *p* < 0.01 compared with the control. This experiment was repeated twice and representative areas are shown.

Involucrin and K10 expressions in the epidermis were decreased when treated with SAHA (Figure 5A). In the image analysis, the calculated stained areas for involucrin were 49.53 ± 9.72, 41.3 ± 9.13, 5.24 ± 2.34, and 5.47 ± 2.83 in the control, 0.1, 1, and 10 nM SAHA treated samples, respectively (Figure 5B). And the calculated stained areas for cytokeratin 10 were 35.02 ± 5.23, 32.42 ± 6.89, 0.67 ± 0.29, and 0.42 ± 0.23 in the control, 0.1, 1, and 10 nM SAHA treated samples, respectively (Figure 5C).

**Figure 6:**
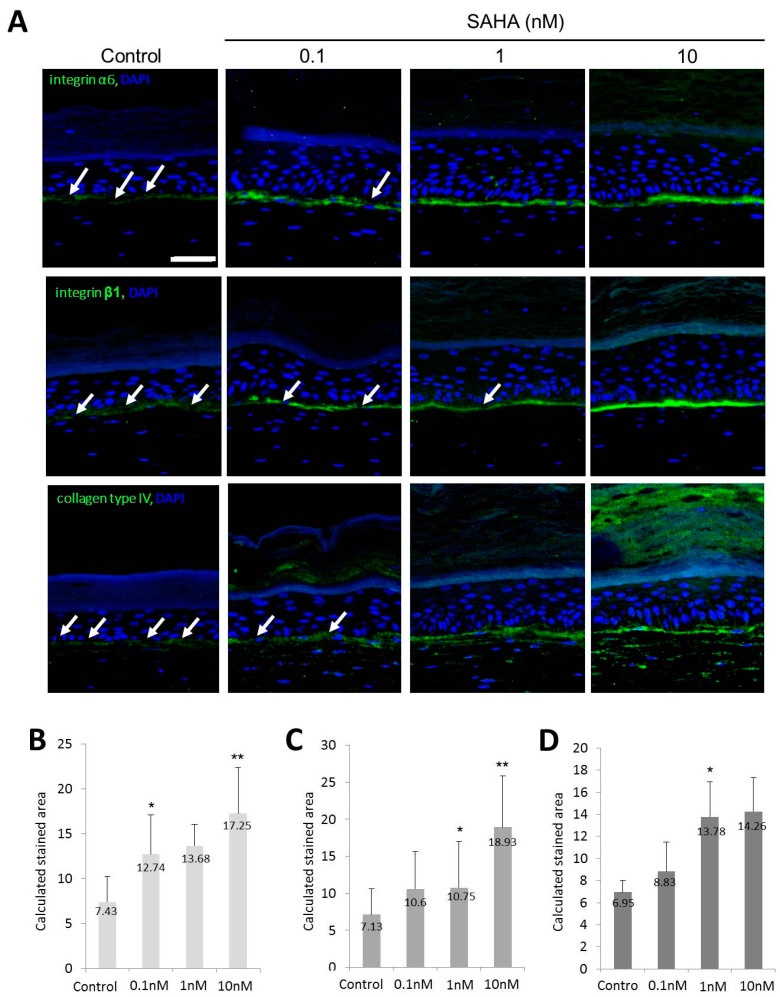
Expression of markers associated with basement membrane in SE. (**A**) Staining of integrin α6, integrin β1, and type IV collagen along the basement membrane in SAHA treated samples. Discontinuity was shown by white arrow (green; integrin α6, integrin β1, and type IV collagen respectively, ×200, scale bar is 50 μm); (**B**) Calculated stained area for integrin α6; (**C**) Calculated stained area for integrin β1 after SAHA treatment; (**D**) Calculated stained area for type IV collagen after SAHA treatment. Six circular areas were selected and the average area stained above a certain intensity was determined. Statistical significance was calculated with the Mann-Whitney-*U* test, * *p* < 0.05 compared with the control, ** *p* < 0.01 compared with the control. This experiment was repeated twice and representative areas are shown.

Furthermore, integrin α6, integrin β1, and type IV collagen in the basement membrane were more strongly expressed in SAHA treated samples (Figure 6A). According to the image analysis, the calculated stained areas for integrin α6 were 7.43 ± 2.87, 12.74 ± 4.39, 13.68 ± 2.39, and 17.25 ± 5.11 in the control, 0.1, 1, and 10 nM SAHA treated samples, respectively (Figure 6B). The calculated stained areas for integrin β1 were 7.13 ± 3.5, 10.6 ± 5.07, 10.75 ± 6.24, and 18.93 ± 6.92 in the control, 0.1, 1, and 10 nM SAHA treated samples, respectively (Figure 6C). The calculated stained areas for type IV collagen were 6.95 ± 1.06, 8.83 ± 2.65, 13.78 ± 3.18, and 14.26 ± 3.05 in the control, 0.1, 1, and 10 nM SAHA treated samples, respectively (Figure 6D). Positively stained areas for integrin α6, integrin β1, and type IV collagen in basal layer were increased in a dose dependent manner (Figure 6B–D).

### 2.3. HDAC1 Expression in Rapidly Adhering Keratinocytes to Type IV Collagen

**Figure 7:**
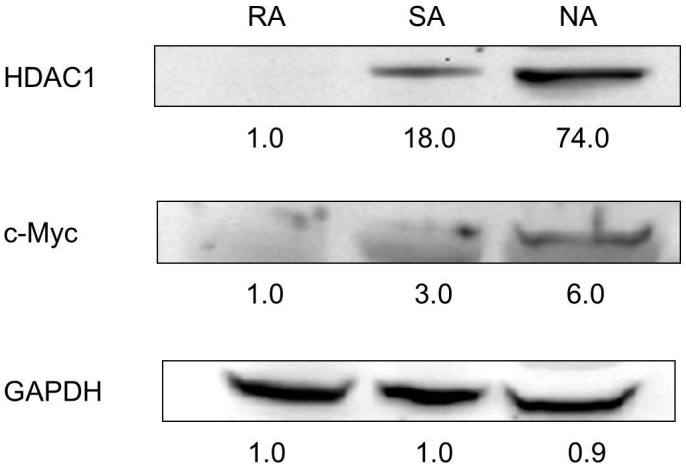
Expression of HDAC1 in three keratinocyte groups. RA, SA, and NA cells were subjected to a Western blot analysis with antibodies against HDAC1 and c-Myc. Equal protein loadings were confirmed using anti-GAPDH antibody. HDAC1 was not expressed in RA cells. HDAC1, histone deacetylases 1, RA, rapidly adhering, SA, slowly adhering, NA, non-adhering.

Three populations of epidermal keratinocytes were isolated in accordance with their ability to adhere to collagen type IV: i.e., rapidly adhering (RA), slowly adhering (SA), and non-adhering (NA) cells. Previously, we have demonstrated that RA cells may represent epidermal stem cells [25]. In the present study, the Western blot analysis showed that RA cells did not express HDAC1, whereas SA cells and NA cells strongly expressed HDAC1 (Figure 7).

## 3. Discussion

Our data show that the expression pattern of p63 combined with HDAC1 could be helpful in identifying the IFE-stem cells. Thus far, several markers have been proposed and used to characterize stem cells in the IFE; however, there has not been a consensus. The transcription factor, p63, was commonly regarded as a potential epidermal stem cell marker. It is known to be essential for epidermal development, proliferation, and asymmetric cell division [17,26]. However, the findings of this study (Figure 1), in accordance with previous studies, [17] show that p63 is expressed not only in the basal layer, but also in the upper layer. This suggests that p63 can also be a marker for proliferating keratinocytes.

HDAC1 was expressed mainly in the upper epidermal layer, where cells go into terminal differentiation (Figure 1). In a previous study, HDAC1 formed a heteromeric nuclear complex with E2F-5 and p130 [24]. The overexpression of E2F-5 specifically inhibited DNA synthesis in undifferentiated keratinocytes, in a HDAC-dependent manner, suggesting that E2F-5·p130·HDAC1 complexes are likely involved in the withdrawal from the cell cycle that responds to differentiation stimuli. In this respect, we can assume that the proliferative potential or “stemness” of basal cells that express HDAC1 is inhibited. In other words, HDAC1-positive cells may be considered as cells that are on the differentiation process. In this study, there was a subpopulation of basal cells that showed a p63-positive/HDAC1-negative staining pattern. We assert that these cells are IFE-stem cells. These cells made up a minority subpopulation, which is one of the important stem cell properties, and dispersed along the basement membrane. Moreover, these cells became less prevalent as age increased. A characteristic feature of skin aging is a decrease in the capacity for wound repair and regeneration. Although the exact mechanism of aging has not yet been fully established, it is widely accepted that senescent changes in stem cells are responsible [27]. This notion may account for the decrease in the portion of epidermal stem cells in the aged group of this study. Further investigations with a large number of cases in each age group would be necessary for confirmation.

SAHA is an inhibitor of HDAC and has been used to treat cutaneous T cell lymphoma [28]. In an SE model, SAHA treatment increased the epidermal thickness, while decreasing the terminal differentiation. In an SE model, basal cells became more cuboidal after SAHA treatment (Figure 3). The number of epidermal cell layers and parakeratosis in the horny layer were also increased in a dose-dependent manner. This suggests that the inhibition of HDAC may induce proliferation and suppress differentiation of the epidermis, which can be interpreted as the possibility of HDAC having an important role in epidermal differentiation. Similar results were produced by an immunohistochemical study. After SAHA treatment, the number of p63-positive cells was notably increased (Figure 4). As you can see in Figure 4, the general pattern of HDAC1 in SE model did not correspond to that in human samples. We think this result comes from the characteristic feature of SE, in that SE is in the status of active proliferation and differentiation at the same time. Meanwhile, expressions of keratinocyte differentiation markers, including involucrin and K10, were significantly decreased in a dose-dependent manner (Figure 5). Finally, the states of the basement membrane were investigated by examining the expression of type IV collagen and integrins. The results showed that SAHA treatment, in a dose-dependent manner, produced stronger expression of type IV collagen with better continuity (Figure 6). The expression of integrin α6 and β1 also showed a marked increase with better continuity in the SAHA-treated samples compared with the control (Figure 6). Type IV collagen is a major component of the basement membrane, and integrins are transmembrane receptors connecting the extracellular matrix of the basement membrane with the cellular membrane of basal cells. In the skin, high integrin levels can distinguish the epidermal stem cells [29]; and now, integrin expression can be used to enrich stem cells in mixed cell populations [30,31]. Type IV collagen and integrins play important roles in shaping the epidermal stem cell niche [8]. Moreover, we have previously demonstrated that the restoration of type IV collagen is important for the maintenance of stem cells in the skin [8]. Furthermore, many evidences indicate that the maintenance of stem-cell properties is highly dependent on integrin adhesion [32]. Taken together and including the results from an SE model, HDAC inhibition promotes epidermal cell proliferation and represses differentiation, which may be interpreted as an increase in the “stemness”. We can presume that HDAC inhibition results from the improvement of niche environment. SAHA strongly inhibits both class I and II HDACs. While class II HDACs are known to be distributed mainly in certain internal organs like the heart, skeletal muscle, brain, etc., class I HDACs containing HDAC1 are known to be distributed ubiquitously, including in skin [33]. They also have been reported to be involved in epidermal development and differentiation [16]. Therefore, we think the effect of SAHA on SE is primarily through the inhibition of class I HDACs.

Previously, we isolated three populations of epidermal keratinocytes according to the collagen type IV: i.e., RA, SA, and NA cells, and showed that RA cells represent epidermal stem cells via FACS analysis, Giemsa staining, electron microscopy, and Western blot [25]. In the present study, RA cells did not express HDAC1 in the Western blot analysis. SA cells and NA cells expressed HDAC1, and the expression was more obvious in NA cells. This pattern was similar to that of c-Myc, which has been known to be important in the conversion of stem cells to TA cells [34], but more obvious (Figure 7). This result lends weight to the idea that HDAC1 is involved in the differentiation process of keratinocytes.

In conclusion, our results suggest that p63-positive/HDAC-negative cells have stem cell properties, whereby a combination of these expressions can be regarded as a potential marker for IFE-stem cells. Further investigations showing co-localization of these cells with other epidermal stem cell markers, or isolating these cells and proving their “stemness” are necessary.

## 4. Experimental Section

### 4.1. Human Samples

Tissue samples from six normal volunteers were collected. The samples were classified into three groups based on age: young age (22 and 25), middle age (43 and 50), and old age (61 and 75). This study was approved by the institutional review board of Seoul National University Bundang Hospital (projection identification code: 02-2014-010, date of approval: 1 August 2014), and informed consent was obtained from all subjects.

### 4.2. Immunohistochemical Staining of Human Samples

Immunohistochemical analyses were performed using formalin-fixed tissues. The paraffin sections were deparaffinized in a HistoChoice Clearing Agent (Amresco, Solon, OH, USA) and rehydrated from graded ethanol to distilled water. Antigen retrieval was performed using Trilogy solution (Cell Marque, Rocklin, CA, USA) and a pressure cooker. Blocking was performed using normal goat (X0907, Dako, Carpinteria, CA, USA) and donkey (ab7475, Abcam, Cambridge, UK) serums. The slides were incubated overnight with primary antibodies, HDAC1(10E2), mouse monoclonal antibody (sc-81598, Santa Cruz Biotechnology, Santa Cruz, CA, USA), P63 (H-137), and rabbit polyclonal antibody (sc-8343, Santa Cruz Biotechnology, Santa Cruz, CA, USA). Secondary antibodies were Alexa Fluor^®^ 488 goat anti-mouse IgG (A11001, Molecular Probes^®^, Invitrogen, Carlsbad, CA, USA) and Rhodamine donkey anti-rabbit (sc-2095, Santa Cruz Biotechnology, Santa Cruz, CA, USA). After staining with DAPI (1 μg/mL, 10236276001, Roche, Indianapolis, IN, USA), images were obtained by Confocal Laser Scanning Microscope (CARL ZEISS, #LSM710, Jena, Germany) and analyzed using ZEN 2011 microscope software (CARL ZEISS). The percentages of epidermal cells with p63-positive/HDAC1-negative staining pattern (DAPI-stained) were obtained and compared between the three age groups.

### 4.3. Cell Culture

The primary cultures of normal human keratinocytes and fibroblasts were isolated from the human foreskins obtained during circumcision [25]. All skin samples were obtained with informed consent. Keratinocytes were cultured in serum-free KGM (keratinocyte growth medium, CC-3111, Lonza, Walkersville, MD, USA), and fibroblasts were cultured in Dulbecco’s modified Eagle’s medium (DMEM; LM001-05, WelGENE, Daegu, Korea) supplemented with 10% fetal bovine serum (FBS; Thermo Scientific HyClone, Logan, UT, USA). The media was changed every 2 or 3 days. All cultures were incubated in a humidified atmosphere containing 5% CO_2_ at 37 °C and were used at the passages 2–4.

### 4.4. Culture of Skin Equivalent and SAHA Treatment

The cytotoxicity of SAHA, (SML0061, Sigma-Aldrich, Oakville, ON, Canada) was tested using a Cell Counting Kit-8 (CCK-8; CK04, Dojindo, Kumamoto, Japan). The cells (3 × 10^3^ fibroblasts or 1 × 10^4^ keratinocytes per well) were seeded into 96-well plates. To test the cytotoxicity to fibroblasts, the full medium was replaced with serum-free DMEM. After 24 h, the cells were treated with SAHA (0.1, 1, and 10 ng/mL) containing serum-free media and incubated for additional 24 h. To test the cytotoxicity to keratinocytes, KGM was removed and KBM (contain SAHA, 0.1, 1, and 10 ng/mL) was added and incubated for 24 h. To measure cell viability, CCK-8 solution was added according to the manufacturer’s instructions, and the cells were incubated for another 2 h at 37 °C. The amount of water-soluble formazan generated by the dehydrogenase activity in the cells was measured by an optical density at 450 nm, using a SpectraMax Plus Microplate Reader (Molecular Devices, Sunnyvale, CA, USA). SEs were constructed following our previous method [25]. In brief, dermal substitutes were prepared according to the method described by Bell (with some modifications) [35]. Type I collagen was extracted from the tendons of rat-tail. Dermal substitutes were then made by mixing eight volumes of type I collagen solution with one volume of 10× concentrated DMEM/F12 (DMEM and Ham’s nutrient mixture F12 at a ratio 3:1; Invitrogen GIBCO, Grand Island, NY, USA) and one volume of neutralization buffer (0.05 N NaOH, 0.26 mM NaHCO_3_, and 200 mM HEPES from Sigma), followed by an addition of 5 × 10^5^ fibroblasts. After gelling in a 25 mm polycarbonate membrane insert (3.0 µm pore size, 137435, Nalge Nunc, Naperville, IL, USA), human keratinocytes (1 × 10^6^ cells) were then seeded onto the dermal substitute. After 1 day, they were cultured at the air-liquid interface for an additional 14 days. The growth medium consisted of 1× DMEM/F12 (DMEM and Ham’s nutrient mixture F12 at a ratio 3:1; WelGENE), supplemented with 5% FBS, 0.4 μg/mL hydrocortisone (Sigma, St. Louis, MO, USA), 1 μM isoproterenol (Sigma), and 5 μg/mL insulin (Sigma). A low concentration of EGF (1 ng/mL; Sigma) was also added to the submerged culture, and a higher concentration of EGF (10 ng/mL) was added to the air-liquid interface culture. The medium was changed three times per week, and all experiments were repeated at least twice under the same condition. SAHA (0.1, 1, and 10 ng/mL) was added nine days after air exposure, as well as at each time when the medium was changed.

### 4.5. Immunohistochemistry of Skin Equivalent (SE) and Image Analysis

After 15 days, SEs were fixed with the solution of 1% paraformaldehyde and 0.1% glutaraldehyde and were embedded in paraffin. Then, each section (3 µm) was stained with hematoxylin and eosin (H&E). To evaluate the change of the number of the keratinocyte layer, three spots were randomly selected in Figure 3, and the mean number of layers was obtained. Remaining procedures, including immunohistochemistry, were performed similarly to the human samples, as previously described. The primary antibodies that recognize p63 (4A4) (GTX23239, GeneTex, Irvine, CA, USA) was used to investigate the changes of the proliferation state. In Figure 4, percentages of epidermal cells with a p63-positive staining were obtained. In addition, involucrin (sc-28557, Santa Cruz Biotechnology, Santa Cruz, CA, USA) and cytokeratin 10 (sc-23877, Santa Cruz Biotechnology, Santa Cruz, CA, USA) were used to check the differentiation state. Finally, integrin α6 (N-19) (sc-6597, Santa Cruz Biotechnology, Santa Cruz, CA, USA), integrin β1 (sc-9970, Santa Cruz Biotechnology, Santa Cruz, CA, USA), and type IV collagen (ab6586, Abcam, Cambridge, UK) were used to figure out the basement membrane state. All images were analyzed using an image analysis program (MetaMorph^®^ Microscopy Automation & Image Analysis Software; Molecular Devices, Sunnyvale, CA, USA). At 200× magnification, six circular areas with the same diameter were selected, and the average areas stained above a certain intensity were determined.

### 4.6. Isolation of Primary Epidermal Cells

Human keratinocytes were isolated and maintained as described above. Epidermal cells were divided into three groups in accordance with their abilities to adhere to type IV collagen as described in a previous study [25]. The cells that have the ability to adhere to type IV collagen within 10 min at 37 °C were selected and classified as rapidly-adhering (RA) cells. The remnant cells were rinsed off and transferred to another culture dish. These non-adherent cells were then maintained for a further 24 h and adherent cells were again selected (slowly-adhering (SA) cells). Then, the non-adherent cells were rinsed off and collected (non-adhering (NA) cells).

### 4.7. Western Blot Analysis

The cells were lysed in a cell lysis buffer (62.5 mM Tris-HCI (pH 6.8), 2% SDS, 5% β-mercaptoethanol, 2 mM phenylmethylsulfonyl fluoride, protease inhibitors (Complete, Roche, Mannheim, Germany), 1 mM Na_3_VO_4_, 50 mM NaF, and 4% Protein cocktail). A spectrophotometer (Nanodrop, ND-1000, Thermo Fisher Scientific, Wilmington, DE, USA) was used for a quantitative analysis. Seventy micrograms of protein per lane was separated by SDS-polyacrylamide gel electrophoresis and blotted onto the PVDF membranes, which were saturated with 5% dried milk in Tris-buffered saline containing 0.4% Tween 20. The blots were then incubated with the appropriate primary antibodies at a dilution of 1:1000, which were then further incubated with horseradish peroxidase-conjugated secondary antibodies. Antibodies against HDAC1 (sc-81598, mouse monoclonal, CA, USA), c-Myc (sc-42, mouse monoclonal), and GAPDH (sc-25778, rabbit polyclonal), were obtained from Santa Cruz Biotechnology (Santa Cruz, CA, USA). Bound antibodies were detected using an enhanced chemiluminescence plus kit (Clarity^TM^ Western ECL Substrate, BIO-RAD, 170-5060, Santa Cruz, CA, USA).

### 4.8. Statistical Analysis

The Kruskal-Wallis test was used to compare the percentages of epidermal cells with p63-positive/HDAC1-negative staining pattern in the human samples. A statistical analysis was performed using Microsoft Excel and SPSS version 20.0 (IBM, Chicago, IL, USA). A *p*-value of less than 0.05 was considered to be statistically significant.

## 5. Conclusions

We showed that there was a minor subpopulation with a p63-positive and HDAC1-negative staining pattern in the basal layer. We also showed that this minor subpopulation decreased with age. An SE model treated with HDAC inhibitor proved that HDAC may be essential for the differentiation of epidermal stem cells. According to our Western blot analysis, RA cells that have been identified to represent epidermal stem cells did not express HDAC1 at all. Therefore, we suggest that p63-positive and HDAC1-negative expressions can be a potential marker for distinguishing epidermal stem cells.
